# Supplementary material for: Antibody Conjugates Bispecific for Pollen Allergens and ICAM-1 with Potential to Prevent Epithelial Allergen Transmigration and Rhinovirus Infection
Source: Int J Mol Sci. 2023 Feb 1;24(3):2725. doi: 10.3390/ijms24032725 (PMC9917280; doi:10.3390/ijms24032725)
Supplement: Supplementary file 1 [file ijms-24-02725-s001.zip › ijms-2089471-supplementary.pdf]

Supplemental tables and figures

Table S1. Inhibition of patients' IgE binding to Phl p 5 and Phl p 2 that had been pre-incubated with Phl p 5- or Phl p 2-specific IgG_1 and Phl p 5- or Phl p 2-specific rabbit serum.

**Phl p 5**						
	**Phl p 5 IgG_1**	**Phl p 2 IgG_1**	**Inhibition % to Phl p 5**	**Phl p 5 rabbit**	**Phl p 2 rabbit**	**Inhibition % to Phl p 5**
Patient 8	1.470	1.388	0.0	0.106	1.595	93.4
Patient 9	0.532	0.601	11.4	0.223	0.666	66.6
Patient 10	0.355	0.366	2.9	0.218	0.371	41.2
Patient 11	0.401	0.443	9.6	0.233	0.472	50.7
Patient 12	0.562	0.640	12.1	0.215	0.679	68.3
**Mean**			7.2			64.0
**Phl p 2**						
	**Phl p 2 IgG_1**	**Phl p 5 IgG_1**	**Inhibition % to Phl p 2**	**Phl p 2 rabbit**	**Phl p 5 rabbit**	**Inhibition % to Phl p 2**
Patient 8	0.222	0.913	75.7	0.278	0.898	69.0
Patient 9	0.340	0.950	64.2	0.401	0.937	57.1
Patient 10	0.238	0.548	56.6	0.275	0.486	43.3
Patient 11	0.467	0.772	39.5	0.326	0.771	57.7
Patient 12	0.633	0.828	23.5	0.316	0.831	62.0
**Mean**			51.9			57.8

ELISA plate-bound Phl p 5 and Phl p 2 had been pre-incubated each with 20-fold excess of non-blocking and blocking allergen-specific monoclonal antibodies (i.e., Phl p 5 IgG_1 and Phl p 2 IgG_1) or polyclonal rabbit sera (Phl p 5 rabbit and Phl p 2 rabbit). Allergens were subsequently exposed to sera of 5 grass pollen allergic patients. OD values corresponding to IgE reactivity to Phl p 5 and Phl p 2 are shown as means of triplets. Reduction of patients' IgE binding to Phl p 5 and Phl p 2 after pre-incubation with non-blocking compared to blocking monoclonal or polyclonal antibodies is given as percentage in the 4th and 7th column.

Table S2. Antibodies and fluorophores used in ELISA, flow cytometry (FC) and immunofluorescence microscopy (IFM).

**Antibody or fluorophore**	**Conjugate**	**Company**	**Clone**	**Cat. no**	**used in**
Goat anti-human IgG F(ab´)2	Alkaline Phosphatase	Thermo Fisher Scientific	polyclonal	31312	ELISA
Rat anti-mouse IgG_1	Alkaline Phosphatase	BD Pharmingen	X56	557272	ELISA
Mouse anti-human IgE	Alkaline Phosphatase	BD Pharmingen		555859	ELISA
Mouse IgG, κ anti-human ICAM-1	Biotin	LifeSpanBioSciences	15.2	LS-C134489	ELISA, FC
Mouse IgG1, κ anti-human IgA_1/A_2 isotype control	Biotin	BD Pharmingen	G20-359	555884	FC
Dy Light 488	Streptavidin	Invitrogen		SE241784	FC

Phl p 5-specific rabbit serum	unconjugated	Charles River	polyclonal		FC
Pre-immune rabbit serum	unconjugated	Charles River	polyclonal		FC
Goat anti-rabbit IgG	Alexa Fluor 405	Invitrogen	polyclonal	A31556	FC
Fixable viability dye	eFluor 780	ThermoFisher Scientific		65-0865-14	FC
Goat anti-mouse IgG	Alexa Fluor 488	Invitrogen	polyclonal	A11001	IFM
Goat anti-rabbit IgG	Alexa Fluor 568	Invitrogen	polyclonal	A11011	IFM
DAPI, 4′,6-diamidino-2-phenylindole, dihydrochloride	unconjugated	Roche Diagnostics GmbH		10236276001	IFM
DRAQ5	unconjugated	ThermoFisher Scientific		62254	IFM

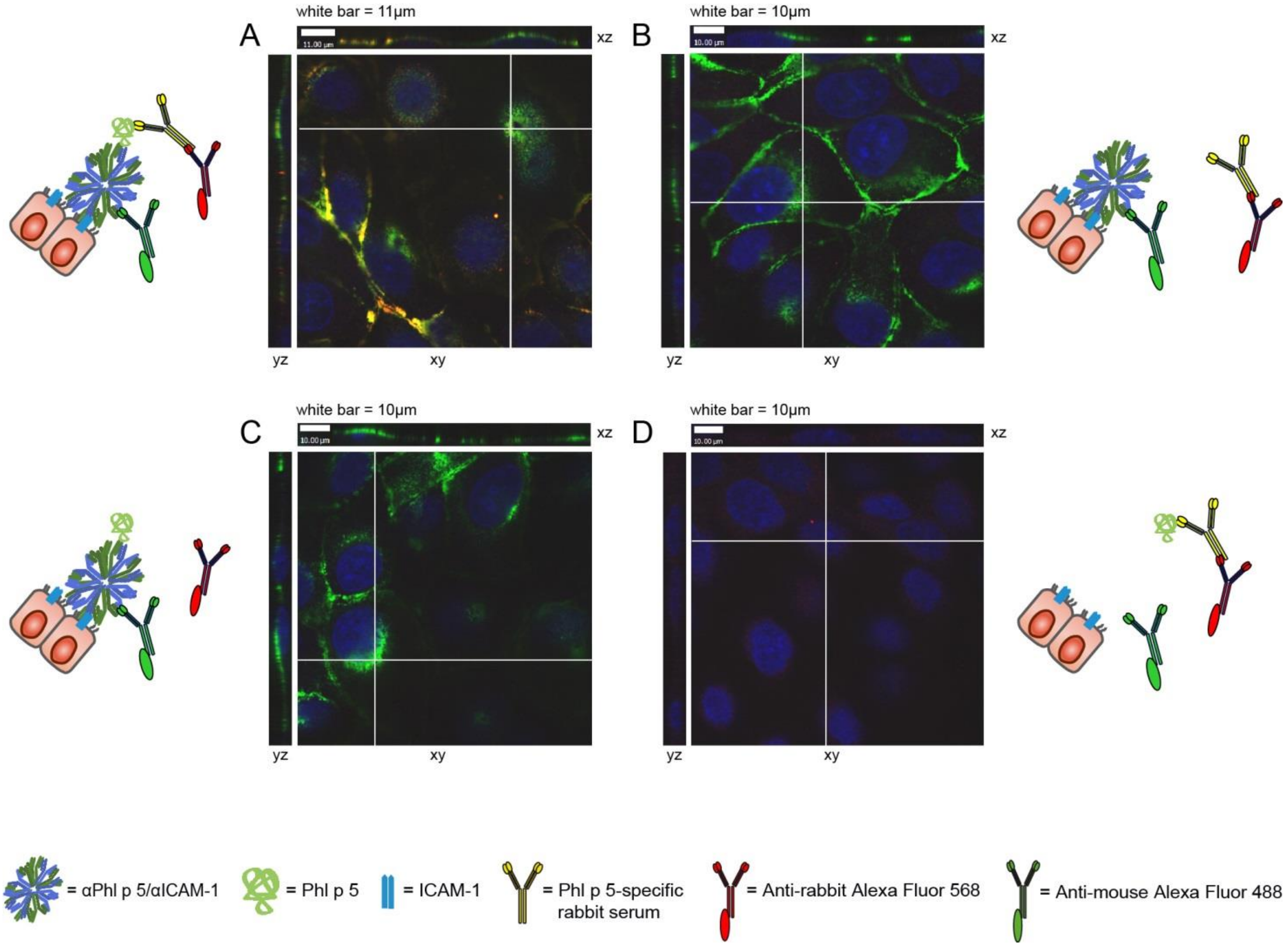

Figure S1. Phl p 5 is captured by surface immobilized αPhl p 5/αICAM-1 conjugates. 16HBE14o- cells were incubated with αPhl p 5/αICAM-1 (**A,B,C**) and/or Phl p 5 (**A,C,D**) and after cell fixation, permeabilized with saponin. Bound conjugates or Phl p 5 were detected with Alexa Fluor 488-labeled anti-mouse antibodies (green) and Alexa Fluor 568-labeled anti-rabbit antibodies (red), respectively. Nuclei were visualized with DRAQ5 (blue), and merged images are shown. Yellow coloring (top left) indicates co-localization of αPhl p 5/αICAM-1 and Phl p 5. Scale bars are 10μm or 11 μm. Confocal microscopy images show xy-axes, xz-axes and yz-axes.
